# Timing of precipitation in an arid environment: Effects on population performance of a large herbivore

**DOI:** 10.1002/ece3.3718

**Published:** 2018-02-22

**Authors:** Levi J. Heffelfinger, Kelley M. Stewart, Anthony P. Bush, James S. Sedinger, Neal W. Darby, Vernon C. Bleich

**Affiliations:** ^1^ Department of Natural Resources and Environmental Sciences University of Nevada Reno Reno NV USA; ^2^ Mojave National Preserve National Park Service Barstow CA USA; ^3^ Sierra Nevada Bighorn Sheep Recovery Program California Department of Fish and Game Bishop California USA

**Keywords:** climate change, drought, juvenile survival, Mojave Desert, normalized difference in vegetation index, *Odocoileus hemionus*

## Abstract

Climate models predict that shifts in temperature and precipitation patterns are likely to occur across the globe. Changing climate will likely have strong effects on arid environments as a result of increased temperatures, increasing frequency and intensity of droughts, and less consistent pulses of rainfall. Therefore, understanding the link between patterns of precipitation, temperature, and population performance of species occupying these environments will continue to increase in importance as climatic shifts occur within these natural ecosystems. We sought to evaluate how individual, maternal, population, and environmental, particularly temperature and precipitation, level factors influence population performance of a large herbivore in an arid environment. We used mule deer (*Odocoileus hemionus*) as a representative species and quantified juvenile survival to test hypotheses about effects of environmental factors on population performance. Precipitation events occurring in mid‐ to late‐pregnancy (January–April) leading to spring green‐up, as indexed by normalized difference in vegetation index, had the strongest positive effect on juvenile survival and recruitment. In addition, larger neonates had an increased probability of survival. Our findings indicate that timing and amount of precipitation prior to parturition have strong influences on maternal nutritional condition, which was passed on to young. These results have important implications for understanding how animal populations may benefit from timing of precipitation during spring and prior to parturition, especially in arid environments.

## 1 INTRODUCTION

Large‐scale climate models predict that increasing fluctuations in temperature and timing of precipitation are likely to occur across the globe (Meehl et al., 2000). The magnitude of these changes is expected to vary throughout latitudes and more specifically, between ecosystems. Our changing climate likely will have especially strong effects on arid environments as a result of increasing frequency and intensity of droughts, shifts in timing and intensity of precipitation, and increasing temperatures. In middle latitudes, which include the deserts of the southwestern United States, precipitation events are generally expected to become sporadic and intense when they occur (Hennessy, Gregory, & Mitchell, 1997). Temporal variation in precipitation from the historical norm also is expected to occur in annual rainfall patterns (Karl & Knight, 1998). Concurrently, temperatures are expected to rise beyond the typical maximums already exhibited in these hot, dry environments (Mahlman, 1997; Meehl et al., 2000). Shifts in temporal variation of precipitation events coupled with higher temperatures are predicted to increase frequency and intensity of droughts in these ecosystems, which may have substantial effects on biota inhabiting, and already well‐adapted to, these arid regions (Seager et al., 2007).

Arid environments frequently exhibit high ambient daytime temperatures, sporadic precipitation, and spatially or temporally sparse nutrients. Animals cope with high ambient temperatures through many anatomical, physiological, and behavioral processes, such as increased peripheral blood flow, long appendages, and various methods of evaporative cooling while minimizing water loss in arid environments (Cain, Krausman, Rosenstock, & Turner, 2006; Hales, 1973). The limited amount of annual precipitation in arid environments likely has a strong influence on life history characteristics, physiology, and species composition of both plants and animals inhabiting those ecosystems (Chesson et al., 2004). Precipitation pulses not only provide animals with temporary, yet essential, sources of free‐standing water, but also drive forage abundance and forage quality in these ecosystems (Marshal, Krausman, & Bleich, 2005). Vegetation in arid environments, especially in the Mojave Desert, has adapted to respond rapidly to rainfall with increased productivity (Ackerman & Bamberg, 1974). Concurrently, animals have also adapted to high temporal variation of short‐lived, rapidly responding nutritional resources and the availability of water from precipitation.

Large, herbivorous mammals can be found in almost all ecosystems ranging from the arctic tundra, to deserts, to the tropical rainforests (Nowak, 1999). In arid environments with widely distributed resources and lower rates of plant productivity, large herbivores require large home ranges to meet nutritional and, thus, energetic demands to maintain viable populations (Heffelfinger, 2006). In some areas, large herbivores expand their home ranges during the hot‐dry seasons to include permanent water sources because of the lack of preformed water in biomass of forage plants (Marshal et al., 2006). Quality of forage plants in arid environments increases as a function of timing and amount of precipitation. Desert plants respond to pulses of precipitation by exhibiting increased growth, biomass, water content, crude protein content, and digestibility (Marshal et al., 2005). Thus, when plant production is high in arid environments, home range sizes may decrease with greater abundance of resources, resulting in decreased expenditure of energy because of the overall greater quality and quantity of available forage (Heffelfinger, 2006). Therefore, quantifying and understanding how environmental factors affect large mammal populations in an already arid ecosystem provide insight regarding the potential effects of a changing climate.

Population performance is directly linked to demographic parameters such as adult survival, reproduction, and recruitment of young into the breeding population. Many factors affect population performance of large mammals including, but not limited to, climate, forage quality, predation, nutrition, human interactions, density dependence, and habitat fragmentation (Parker, Barboza, & Gillingham, 2009; Sinclair & Krebs, 2002; Sinclair, Mduma, & Brashares, 2003). Large mammals, particularly herbivores, typically exhibit high and stable adult survival throughout many environments (Gaillard, Festa‐Bianchet, Yoccoz, Loison, & Toigo, 2000; Hurley et al., 2011; Monteith et al., 2014). Additionally, previous investigators have shown pregnancy rates and fetal rates to be high and consistent (Andelt, Pojar, & Johnson, 2004; Bishop, White, Freddy, Watkins, & Stephenson, 2009). Therefore, juvenile survival and recruitment may be the best indicators of population performance (Gaillard, Festa‐Bianchet, & Yoccoz, 1998).

We used mule deer (*Odocoileus hemionus*) as a representative species to explore how environmental, population‐level, and individual factors affect demographic rates of a large herbivore in an arid ecosystem. Mule deer occur throughout most of North America, including the arid ecosystems of the southwestern United States (Heffelfinger, 2006), illustrating a high degree of adaptability to widely disparate climatic conditions. This variation in habitat use allows investigators to evaluate population responses to many different environmental factors. Similar to other large herbivores, adult mule deer exhibit high and stable survival rates throughout most of their geographical range (Bender, Hoenes, & Rodden, 2012; Bishop et al., 2009; Hurley et al., 2011; Monteith et al., 2014). Given the importance of the influence of juvenile survival on population performance (Gaillard et al., 1998), our objectives were to evaluate and quantify factors influencing juvenile survival in an arid environment. We hypothesized that neonatal survival is strongly affected by fluctuating environmental variables frequently exhibited in desert ecosystems. Therefore, we focused on assessing the effects of amount and timing of precipitation in conjunction with vegetation green‐up on juvenile survival.

## 2 MATERIALS AND METHODS

### 2.1 Study system

**Figure 1:**
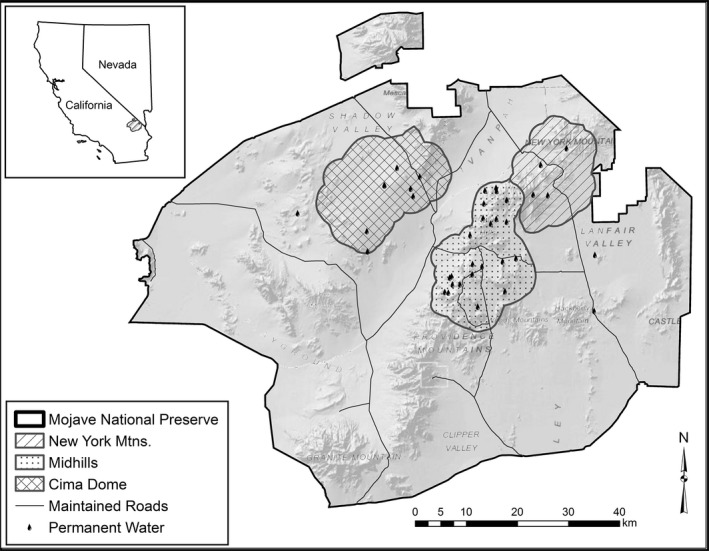
Map of the Mojave National Preserve, CA, USA, with study area delineations representing habitat types and permanent water sources exhibited in the Mojave Desert. Inset map shows location relative to Nevada and California, USA (shaded). Figure recreated from McKee et al. (2015)

We conducted our research in Mojave National Preserve, located in San Bernardino County, in southeastern California, USA (35°00′N 115°28′W; Figure 1). Mojave National Preserve ranges in elevation from 270 m in the valley bottoms to 2,417 m at the summit of Clark Mountain and covers nearly 650,000 ha (McKee et al., 2015). Mojave National Preserve provides an excellent area for studying organisms occupying arid environments because of its vast size, and inclusion of three of the four desert ecosystems in North America: the Mojave, Sonoran, and Great Basin deserts (National Park Service, 2017). The area is composed of extensive valleys separating rugged mountain ranges composed of granite, basalt, and igneous rock (McKee et al., 2015).

Weather patterns in Mojave National Preserve are similar to the majority of southwestern deserts, characterized by overall limited precipitation, and relatively higher temperatures. Precipitation patterns are bimodal with peaks during the summer and winter months (McKee et al., 2015). Mean annual precipitation at low elevations is 9.3 cm (1980–present, Soda Springs, northern Mojave National Preserve) and 18 cm at mid‐ to upper‐elevations (2007–2014, Meso West Weather Station, University of Utah, Salt Lake City). Mean maximum temperatures during the summer are 40.5 and 33°C and during the winter are 19 and 13°C at low and high elevations, respectively.

Vegetation communities in the Mojave National Preserve vary by elevation and associated temperature and precipitation (National Park Service, 2017). Lower elevations are dominated by creosote bush (*Larrea tridentata*) and white bursage (*Ambrosia dumosa*) with limited amounts of grasses and forbs (McKee et al., 2015; Thorne, Prigge, & Henrickson, 1981). Mid‐elevations below 1600 m consist of varieties of desert yucca (*Yucca* spp.), blackbrush (*Coleogyne ramosissima*), cholla cactus (*Opuntia* spp.), barrel cactus (*Ferocactus* spp.), and Joshua tree (*Yucca brevifolia*) woodlands while mid‐elevations above 1600 m are dominated by Great Basin sagebrush (*Artemisia tridentata*), Utah juniper (*Juniperus osteosperma*), and pinyon pine (*Pinus monophylla*) (Thorne et al., 1981). Elevations above 1,800 m support remnant patches white fir (*Abies concolor*) in the Clark Mountain Range and New York Mountains (McKee et al., 2015; Thorne et al., 1981).

Mule deer in the Mojave Desert are similar in their reproductive biology to those studied in Round Valley, CA by Monteith et al. (2013). The mating season occurs in the autumn, usually early November and trails off through late December (Heffelfinger, 2006; Wallmo, 1981). Gestation in mule deer is about 200 days (Heffelfinger, 2006; Wallmo, 1981), and the period of parturition in our study area generally extends from mid‐to‐late May through June. Because resources are generally available during winter, and little to no snow is observed, overwinter mortality is generally low. Therefore, in this ecosystem, we consider those young that have survived to 120 days to be recruited into the population.

Three study sites were established based on home ranges of collared deer from a previous study by McKee et al. (2015) that best described suitable mule deer habitat for the area (Figure 1). Cima Dome contains seven permanent water sources within a 40,404 ha area, has little elevation change, and is dominated by Joshua tree woodland with sparse patches of juniper. The Midhills study site contains 19 water sources within a 39,368 ha area and experienced an extensive wildfire in 2005. The burned portion of the study site is dominated by globemallow (*Sphaerelcea* spp.), bitterbrush (*Purshia glandulosa*), and desert almond (*Prunus fasciculata*) within rolling hills but still supports patches of Great Basin sagebrush and pinyon‐juniper woodland at upper elevations in areas that did not burn (McKee et al., 2015). The New York Mountains study site has four water sources within a 27,195 ha area, consists of steep, rocky pinyon‐juniper woodland in the upper elevations, and yucca desert scrub in the lower elevations (Figure 1; McKee et al., 2015).

### 2.2 Data collection

From 2013 to 2016, we captured adult female deer in late February or early March via a net gun fired from a helicopter (Krausman, Hervert, & Ordway, 1985). Captured deer were flown to a central processing station established for each study area where they were fitted with GPS (Global Positioning System) collars (Advanced Telemetry Systems, G2110D, Isanti, MN USA) and uniquely marked with ear tags. Collars were equipped with a mortality sensor and a Very High Frequency (VHF) transmitter. Additionally, collars were programmed to collect one GPS location every 90 min and to drop off approximately 1 year following deployment (McKee et al., 2015). We determined nutritional condition of adult females using standard protocols developed and validated for mule deer (Monteith et al., 2013). Those methods used measurement of subcutaneous fat on the rump using ultrasonography, as well as palpation at rump, ribs, and withers to determine a body condition score (Cook, Stephenson, Myers, Cook, & Shipley, 2007; Monteith et al., 2013; Stephenson, Bleich, Pierce, & Mulcahy, 2002). Pregnancy status also was determined using ultrasonography (Stephenson et al., 1995). Pregnant individuals were then fitted with a vaginal implant transmitter (VIT) that was equipped with both a temperature and a photo sensor (Advanced Telemetry Systems, M3930L; Bishop et al., 2007). Individuals captured nearby were then released from the central processing station or flown back to the original capture location if they were captured over 8 km from the processing station.

Vaginal implant transmitters used during this study were similar to those described by (Bishop et al., 2011). In addition to the temperature sensor traditionally equipped in VITs used in previous studies (Bishop et al., 2007; Carstensen, DelGiudice, & Sampson, 2003; Johnstone‐Yellin, Shipley, & Myers, 2006), VITs in this study also had a programmed photo sensor. This design allowed for VIT expulsion to be detected at night or during the day despite high ambient temperatures in our study site. When a VIT was expelled, a preprogrammed Precise Event Timing (PET) coding was emitted once per minute within the VHF pulses to aid in the identification of expulsion time. The PET coding indicated the time since expulsion in 30‐min increments up to 5 days (Advanced Telemetry Systems, 2017; Bush, 2015), thereby allowing age of the neonate to be calculated precisely.

For clarification, we use the term neonate to describe an offspring in the first week of life and the term young or juvenile for all ages thereafter. Ground crews monitored females outfitted with VITs from 1 May until parturition. Monitoring occasions were <3 days apart for each female (usually every day) to capture neonates as close to parturition as possible or to prevent neonate mortality occurring prior to capture. If PET coding (i.e., the neonate's age) was less than 3 hr, technicians would allow time for critical bonding, and colostrum transfers between neonate and mother before approaching (White, Knowlton, & Glazener, 1972). Technicians would systematically search the area using the location of the VIT or the female's location at initial sighting as the beginning of their search radius (Quintana et al., 2016). Search times were restricted to 30 min to reduce the chance of abandonment of the neonate or stress to the female (Livezey, 1990). When neonates were not located during an initial search, ground crews returned the next day to search for the neonate using the same method (Bush, 2015).

In 2013, two of our collared adult females were pregnant but did not receive a VIT because the birth canal was too narrow to successfully insert a VIT. Additionally, we had one adult each year thereafter that exhibited similar morphology. Females that did not receive a VIT were located and checked for parturition by systematically searching for a neonate every 1–3 days (Bush, 2015). In 2013, we monitored 13 adults that had been active collars from 2012 but did not receive a VIT in 2013, using the same method. Throughout the study we also captured neonates from un‐collared individuals displaying signs of having a neonate nearby or opportunistically finding neonates while tracking or monitoring collared adults (Carstensen et al., 2003).

When a neonate was captured, it was immediately blindfolded, placed in a clean cloth bag and weighed to the nearest 0.1 kg using a spring scale (Pesola Scales, Baar, Switzerland). We measured new hoof growth to the nearest 0.1 mm with a digital caliper and measured chest girth and metatarsus to the nearest 0.1 cm. Sex, state of umbilicus, prominent vegetation type at the birth site, handling time, and a GPS location also were recorded. Finally, each neonate was fitted with an expandable VHF radio‐collar (Advanced Telemetry Systems) with the mortality switch set to 6 hr of no movement, and released.

We used several different methods to reduce abandonment caused by handling. All technicians wore nitrile gloves during handling to reduce scent transfer, and all blindfolds and weigh bags were washed in scent‐free detergent after each use and stored in scent‐free bags during transport. Additionally, technicians were required to wash all clothes in scent‐free detergent and to change clothes between capturing neonates from different mothers. We also stored the expandable collars in scent‐free bags containing native vegetation to reduce the amount of non‐native odors being transferred to the neonate (Livezey, 1990).

Age at capture for each neonate was determined using the PET coding recorded from the VIT. Nevertheless, age at capture had to be estimated for individuals caught from unmarked females or in instances where the VIT or PET coding malfunctioned. When monitoring collared females that did not receive a VIT or had a malfunctioning VIT, time and date were recorded on each occasion we encountered those females. We then used behavior of the neonate, condition of umbilicus, hoof appearance, and body size to estimate maximum possible age of those neonates (Haskell et al., 2007; Haugen & Speake, 1958; Monteith et al., 2014).

Survival of each neonate was monitored daily for the first week of life. Following the first week, each young was checked every 1–3 days until the month of August and weekly thereafter until they reached 120 days of age (4 months). We used 120 days as our measure of survival of young, because at 120 days young were completely weaned from mother and fully dependent on water sources and forage (Heffelfinger, 2006; Sadleir, 1980).

All animal capture and handling procedures were approved by the Institutional Animal Care and Use Committee at the University of Nevada, Reno Protocol # 00058 and were within guidelines established by the American Society of Mammalogists for research on wild mammals (Sikes, 2016). We also complied with capture and handling procedures developed by California Department of Fish and Wildlife.

### 2.3 Individual and environmental covariates

We tested several parameters that may have influenced survival of young in our study. First, we created an index of body size for each neonate at initial capture using chest girth and metatarsus in a principal components analysis (PCA; Dunteman, 1989; R 3.3.2, R Core Team). Both morphological variables loaded similarly in the eigenvector for principal component 1 (PC1) 0.707, and PC1 had an eigenvalue of 1.549, which explained 77.4% of the variation in those morphometric measurements. Therefore, PC1 scores for each neonate were used as an index of body size. We performed a linear regression of body weight against the PC1 scores (*R* ^2^ = .45, *p *<* *.0001) with the residuals used as an index of body condition of young. Positive residuals were considered above average condition given its size, while negative residual values were indicative of poorer condition of neonates at initial capture (Bush, 2015; Jakob, Marshall, & Uetz, 1996; Schulte‐Hostedde, Zinner, Millar, & Hickling, 2005).

We created a covariate representing timing of birth relative to the median parturition date for the given year of the study. We also included additional covariates that were descriptive of the neonate including sex, handling time during capture, and a binomial covariate indicating whether the neonate was one of a set of twins or triplets. We also included covariates describing nutritional condition of the mother at capture, which included body condition score and maximum depth of rump fat from ultrasonography, described previously.

We created a series of covariates based on location data from the GPS radio‐collar of the mother and location data from the birth site obtained from ground crews following capture of neonates. We considered birth site covariates including relative distance from water, distance from roads, and the amount of shrub cover at the birth site to determine if the spatial location of the birth site affected survival of young. We obtained vegetation layers including shrub cover (LANDFIRE, 2014) and used ArcGIS (Version 10.3) to extract values of relative shrub cover and distance to water that the female occupied while the young was known to be alive and with the mother. The series of extracted values were averaged to create individual covariates for shrub cover and distance to water prior to mortality or weaning of young (Table 1). All numerical covariates were converted to standard normal variables prior to model evaluation (Cooch & White, 2017; Zar, 2010).

**Table 1:** Definitions of parameters used to in models used to estimate factors influencing survival of 110 neonatal mule deer captured between 2013 and 2016 on the Mojave National Preserve, CA, USA

Parameters	Definition	Effect type
Year	Year of study (2013–2015 relative to 2016)	Group
Site	Study sites New York Mtns. and Midhills relative to Cima Dome	Group
Precipitation^a^	Mid‐pregnancy cumulative precipitation (January–April)	Group
NDVI^b^	Mid‐pregnancy average Normalized Difference Vegetation Index (February–May)	Group
Age	A specified trend of survival dependent on age	Group
Twin	Whether the juvenile was part of a set of twins or triplets	Group
Sex	Sex of juvenile	Group
Body size	Index of body size of young from Principal Components Analysis	Covariate
Birth date	Birth date relative to median parturition time, annually	Covariate
Juvenile BC	Residuals from regression of juvenile weight given size index	Covariate
Maternal BCS	Body condition score of maternal female	Covariate
Maternal rump fat	Rump fat measurement of maternal female	Covariate
Max temperature	Maximum daytime temperature juvenile was subjected to (time varying)	Covariate
BS paved road^c^	Distance of juvenile's birth site to nearest paved road	Covariate
BS shrub cover^c^	Relative shrub cover measurement of juvenile's birth site	Covariate
BS dirt road^c^	Distance of juvenile's birth site to nearest dirt road	Covariate
BS elevation^c^	Elevation measurement of juvenile's birth site	Covariate
BS water^c^	Distance of juvenile's birth site to nearest permanent water source	Covariate
Distance to water	Average distance to water source of maternal female while juvenile was alive	Covariate
Shrub cover	Average shrub cover occupied by maternal female while juvenile was alive	Covariate

a Mid‐pregnancy precipitation was used as an annual effect and thus could not be modeled with a delineation of year.

b Mid‐pregnancy NDVI values were used as a study site effect and thus could not be modeled with a delineation of study site.

c Birth site covariates were restricted to the first week of life during the model‐building procedure.

We considered several environmental covariates relating to temperature, precipitation, and greenness of vegetation that may affect survival of young. First, we created a time‐varying individual covariate of maximum daytime temperature from birth to 120 days for each individual young. Within each study area, we generated 10 random points throughout the site. We then extracted estimated maximum daily temperature data (TMAX) from the UI METADATA dataset throughout the duration of the study for each random point (Climate Engine, 2017). The series of maximum temperature values were then averaged to create a maximum daily temperature to characterize each study area throughout the investigation. Each individual was assigned the maximum daytime temperature for the area that they occupied, daily, for the duration of their life. Thus, this individual time‐varying covariate was used to test how maximum temperature affected survival of young. We also directly tested how rainfall patterns influenced juvenile survival probabilities within our system, a priori. Data were retrieved from the Mid Hills Station operated by the Western Regional Climate Center, in the center of our Midhills study area, and were assumed to be representative of rainfall conditions of all our study areas. We summed total rainfall within each calendar month. We then evaluated various combinations of monthly sums of total rainfall within and among the trimesters of pregnancy for mule deer in our study area. We started with seasonal groupings of months identified by McKee et al. (2015). We then expanded our modeling to align with biologically meaningful time periods (i.e., pregnancy trimesters or typical rainfall patterns). Our a priori models of monthly rainfall during gestation showed that total rainfall from January through April best‐explained juvenile survival over all other temporal models of biologically meaningful periods of rainfall (ΔAIC_c_ = 1.61).

Lastly, we extracted normalized difference in vegetation index (NDVI) values to further test for the general effect of precipitation on nutritional resources available to pregnant females and thus, juvenile survival in our analysis (Climate Engine, 2017). Within each study area, we randomly selected 10 points to collect NDVI values. We were careful to exclude and resample points to minimize bias in NDVI values from nonfluctuating greenness in certain vegetation types (i.e., Pinyon‐juniper) and effects of north‐ or south‐facing slopes. None of our NDVI images were confounded with atmospheric obstructions and thus, we did not have to remove any of them prior to analyses. When rainfall patterns and NDVI were plotted together, our data indicated a 30‐day lag in green‐up following a rainfall pulse. Thus, we tested average February through May NDVI values in our models. We averaged NDVI values from February to May from those 10 randomly selected points for all processed satellite images within each study area and year to create an overall index of vegetation “greenness” for the site. The NDVI timeframe model outperformed the best precipitation timeframe model (ΔAIC_c_ = 3.81) and was used thereafter as a covariate in survival analyses. All environmental covariates were converted to standard normal variables prior to modeling (Cooch & White, 2017; Zar, 2010).

### 2.4 Survival analysis

We assessed daily survival probabilities of young by study area and among years using the nest survival module in Program MARK (Version 8.0; White & Burnham, 1999; Cooch & White, 2017). The nest survival allows for inconsistent monitoring events, which frequently occur in telemetry‐based survival studies (Blomberg, Sedinger, Gibson, Coates, & Casazza, 2014; Dinsmore, White, & Knopf, 2002). We evaluated survival to 120 days of age and created encounter histories based on age at which the young was captured, heard alive via VHF signal, or determined to be dead during the 4‐month period. One adult was killed by a vehicle in 2013 shortly after parturition, which led to a juvenile mortality, and we observed one juvenile mortality related to maternal abandonment in 2015. Those neonates were censored from the survival analysis.

We employed an iterative approach to model selection (Burnham & Anderson, 2002) and first created models to test for annual or study site differences in survival of young. Then, using the top model from this step, we tested various age trends that allowed survival to vary for each day, each week, and all possible combinations that potentially were biologically relevant. Trend models of this type require fewer parameters to estimate and evaluate whether neonate survival varied with age (Bishop et al., 2007).

After identifying the top model including a year‐ or site effect and the appropriate age trend, we began evaluating individual, maternal, population, spatial (birth site characteristics), and environmental covariates (temperature, precipitation, NDVI). We evaluated collinearity among those covariates using a correlation matrix (R 3.3.2 R Core Team). None of our covariates were correlated >0.65; therefore, we considered all predictor variables in candidate models. We realize the several of the variables (e.g., body size and NDVI) were estimates used in the survival analysis, and that in a maximum‐likelihood framework, our estimated covariates may result in overly precise parameter estimates, and may have introduced some additional error into our analysis. At this point, this problem is difficult to resolve in a maximum‐likelihood framework, but also likely results in increased variation among individuals. Therefore, we feel that our analysis and models are appropriate even if there is some increased variation among individuals, resulting from those estimated covariates in the survival analysis.

We employed a sequential model‐building procedure to evaluate hypothesized biological processes (i.e., individual, maternal, population, spatial, and environmental factors) while minimizing the total number of models considered (Blomberg, Sedinger, Nonne, & Atamian, 2013). Precipitation data were available from only the single weather station in the Midhills; as a result, only annual variation could be tested. Additionally, NDVI data were constrained to study site and year, so they were treated as a site by year effect. Covariates for birth site characteristics were constrained to the first week of life because neonates become much more mobile and adults moved away from the vicinity of the birth sites thereafter (White et al., 1972).

Competitiveness for each hypothesis (model) was assessed using the Akaike information criterion adjusted for small sample size (AICc), associated 95% confidence intervals, Akaike weights, and parameter estimates (Anderson, Burnham, & Thompson, 2000; Buckland, Burnham, & Augustin, 1997). The model with the lowest AICc value was considered the most parsimonious model (Burnham & Anderson, 2002). Parameter estimates for covariates were considered statistically significant if 95% confidence interval did not overlap zero. Those parameters that were most influential (i.e., larger effect sizes) indicated which factors were most associated with survival rates of young mule deer. Parameters most predictive of survival of young were then back‐transformed on the logit scale to evaluate the relative effect of each covariate probability of survival, with standard errors estimated via the delta method (Cooch & White, 2017).

## 3 RESULTS

From 2013 to 2016, we captured 119 adult female mule deer throughout all of our study sites. Average body condition score for adult females during the study was 2.22 (*SD* = 0.45), and mean depth of rump fat was 0.14 cm (*SD* = 0.17; Table 2). During the course of our study, we observed three mortalities of collared adult females in 2013, and one in 2014. Overall pregnancy rate was 0.97, and over the course of the study we observed five still births, and captured one set of triplets, 25 sets of twins, and 57 singletons. We captured and collared 37 neonates in the New York Mountains, 39 in the Midhills, and 34 in the Cima Dome study sites. Overall we captured 85 neonates from adults with VITs, 12 from adults with collars only, and 13 from noncollared individuals. Of neonates captured, 62 were male while 58 were female. Parturition dates ranged from 11 May to 26 June throughout the study. Median parturition date varied annually (2013 = 6 June, 2014 = 28 May, 2015 = 3 June, and 2016 = 25 May), with an overall median date of 2 June. We captured 92% of our neonates prior to 4 days of age, and the oldest young captured was 7 days old.

**Table 2:** Descriptive statistics for continuous variables used in analysis of survival of 110 neonatal mule deer on the Mojave National Preserve, CA, USA, between 2013 and 2016

Parameters	Mean	*SD*
Maternal body condition score	2.22	0.45
Maternal rump fat	0.14 cm	0.17
Birth site distance to paved road	5,291 m	3,087
Birth site distance to dirt road	1,236 m	893
Birth site shrub cover	25.91%	19.40
Birth site elevation	1,572 m	101
Birth site distance to water	2,181 m	1,032
Adult female distance to water	2,012 m	902
Adult female shrub cover	26.66%	15.02

See Table 1 for parameter definitions.

**Figure 2:**
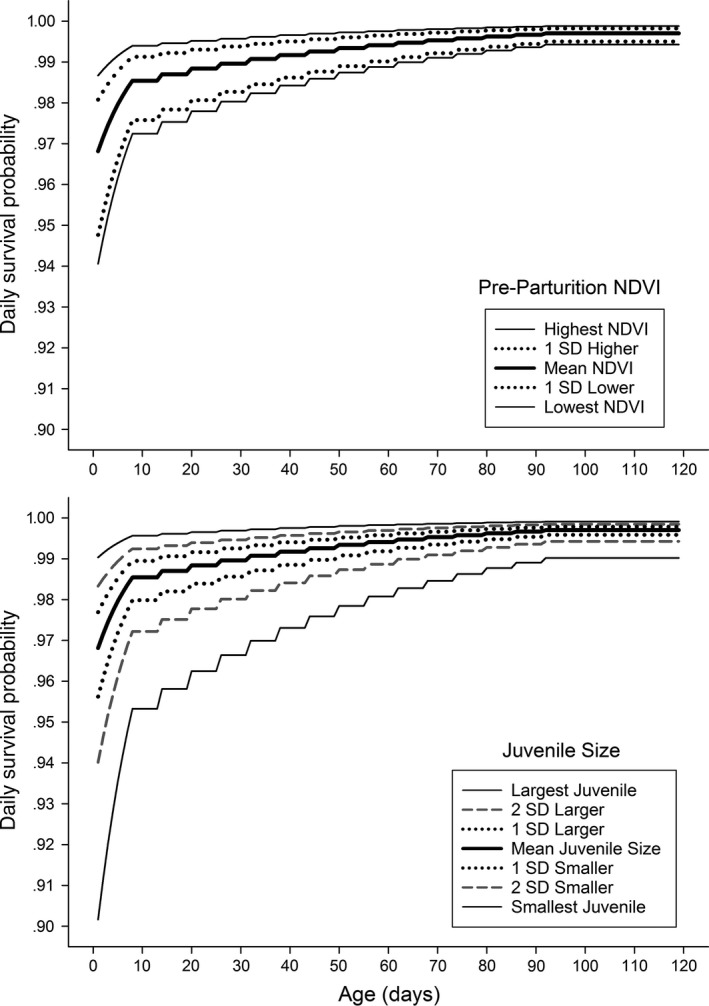
The effect of preparturition Normalized Difference Vegetation Index (NDVI; top) and juvenile size (bottom) on daily survival probability for 110 neonatal mule deer captured on the Mojave National Preserve, CA, USA, between 2013 and 2016. Back‐transformed beta estimates from our top competing model are used to model the effect of variables on daily survival probability throughout the first 120 days of life of a neonate

**Figure 3:**
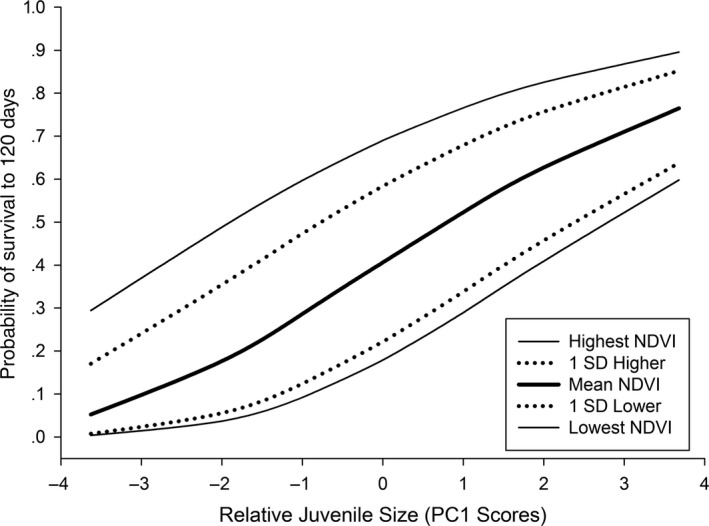
Effect of Normalized Difference Vegetation Index (NDVI) and relative body size of juveniles on estimated probability of survival to 120 days (weaning) for 110 neonatal mule deer captured on the Mojave National Preserve, CA, USA, 2013–2016. Back‐transformed beta estimates from our top competing model are used to model survival to 120 days of life

We found no support for variation in survival among years, study areas, or a combination of the two during our model‐building procedure (ΔAIC_c_ = 41.42, 57.71, and 42.70, respectively). Our most supported (based on AICc scores) survival trend with juvenile age indicated daily survival estimates to 7 days, weekly estimates to 90 days, and a constant survival estimate from 91 to 120 days (Figure 2). Our best‐supported model included that positive trend of survival with age, effects of preparturition NDVI values, and body size (Tables 3 and 4). Mid‐pregnancy NDVI values for each study site and year positively influenced survival (ß = 0.5184 95% CI = 0.2659–0.7709; Figures 2 and 3) and were best supported during our model‐building procedure. Lastly, our index of body size (PC1) of neonates positively influenced survival (ß = 0.3304 95% CI = 0.1009–0.5598; Figures 2 and 3).

**Table 3:** Results from nest survival modeling in program MARK for survival of 110 neonatal mule deer captured on the Mojave National Preserve, CA, USA, from 2013 to 2016

Survival model parameters	ΔAIC_c_	*w* ^i^	*K*	Dev
Age + NDVI + body size	0.00	0.56	4	540.78
Age + NDVI + body size + NDVI*body size	2.00	0.21	5	540.78
Age + NDVI + handling time	4.22	0.07	4	545.00
Age + NDVI	6.31	0.02	3	549.09
Age + NDVI + BS distance to water	7.02	0.02	4	547.81
Age + NDVI + birth date	7.18	0.02	4	547.96
Age + NDVI + juvenile BC	7.41	0.01	4	548.19
Age + NDVI + maternal BCS	7.56	0.01	4	548.34
Age + NDVI + maternal rump fat	7.70	0.01	4	548.48
Age + NDVI + twin	7.99	0.01	4	548.77
Age + NDVI + max temperature	8.02	0.01	4	548.79
Age + NDVI + BS distance to paved road	8.04	0.01	4	548.82
Age + NDVI + sex	8.17	0.01	4	548.95
Age + NDVI + BS shrub cover	8.27	0.01	4	549.05
Age + NDVI + shrub cover	8.27	0.01	4	549.05
Age + NDVI + BS distance to dirt road	8.30	0.01	4	549.08
Age + NDVI + BS elevation	8.31	0.01	4	549.09
Age + NDVI + distance to water	8.31	0.01	4	549.09
Age	21.37	0.00	2	566.15
NDVI	34.10	0.00	2	578.89
Precipitation	37.92	0.00	2	582.70
Year	41.42	0.00	4	582.20
Site*year	42.70	0.00	6	579.48
Null	54.30	0.00	1	601.09
Site	57.71	0.00	3	600.49

Models were created in sequential model‐building procedure while testing viable biological processes and maintaining minimal total models. Models were evaluated and compared using Akaike Information Criterion adjusted for small sample size (AIC_c_). See Table 1 for parameter definitions.

**Table 4:** Parameter estimates and 95% confidence intervals for our most parsimonious model of survival of young on the Mojave National Preserve, CA, USA, 2013–2016

Covariate	β	Lower 95% CI	Upper 95% CI
Intercept	3.30	2.74	3.86
Age trend	0.11	0.07	0.16
NDVI	0.52	0.27	0.77
Body size	0.33	0.10	0.56

The estimated age trend was estimated daily survival to 7 days, weekly survival to 90 days, and then constant survival from 91 to 120 days at which young were considered weaned from mothers.

**Figure 4:**
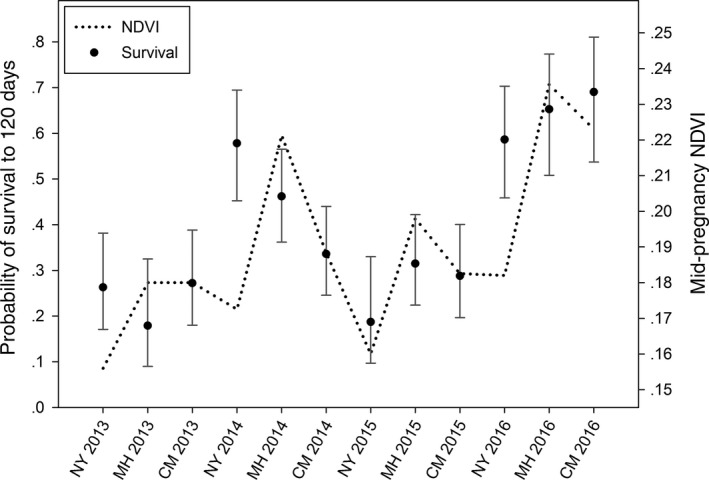
Estimated survival from nest survival analysis in program MARK of 110 neonatal mule deer captured between 2013 and 2016 on the Mojave National Preserve, CA, USA. Estimates are separated by study area and year for the duration of the study (NY, New York Mountains; MH, Midhills; CM, Cima Dome) with 95% confidence intervals indicated in gray. The best‐performing predictor of survival, mid‐pregnancy Normalized Difference Vegetation Index observed for each study area and time period, is represented by the black dotted line

Overall estimated survival of young to weaning (120 days), assuming mean NDVI and body size, was 40.63% (*SE* = 0.05; Figure 3). Back‐transformed estimates from our top model allow us to evaluate the relative effect of each parameter on juvenile survival. NDVI had a positive influence on survival with an estimated survival to weaning of 69.01% (*SE* = 0.07) at the highest NDVI values experienced by pregnant females during the study, and an estimated survival of 17.89% (*SE* = 0.06) at the lowest values (juvenile size held at mean; Figure 3). Additionally, juvenile size positively influenced survival with an estimated survival to 120 days of 76.48% (*SE* = 0.10) for the largest neonates captured during the study versus an estimated survival of 5.24% (*SE* = 0.06) for the smallest (NDVI held at mean; Figure 3). An interaction between juvenile size and preparturition NDVI values was not supported in our modeling procedure (ß = 0.0067 95% CI = −0.2116 to 0.2250). Back‐transformed estimates, however, can be used to evaluate the effect of both parameters simultaneously. Estimated survival to weaning for a juvenile born at the smallest size in a year from a mother that was subjected to the lowest NDVI values was 0.38% (*SE* = 0.01), while a juvenile born at the largest size in a year from a mother exposed to the highest NDVI had an estimated survival of 89.57% (*SE* = 0.05; Figure 3). Our top model estimated juvenile survival (as an effect of NDVI values in each study area and year) ranging from 17.89% (*SE* = 0.06; Midhills, year 2013) to 69.05% (*SE* = 0.07; Cima, Dome year 2016; Figure 4).

## 4 DISCUSSION

Our evaluation of juvenile survival in a population of mule deer occupying an arid environment revealed the importance of preparturition nutritional resources on population performance. Our measured NDVI values for the space occupied by pregnant females from February to May, occurred during the 2nd and 3rd trimesters of pregnancy for most individuals, and had a strong effect on survival of young. The strong evidence that mid‐pregnancy NDVI values positively influenced juvenile survival likely reflects forage quality and quantity available to the mothers following precipitation events in an arid environment (Marshal et al., 2005). Development of the fetus is at a crucial point during that time period because nutritional condition of the neonate at birth is directly correlated with available nutrients and condition of the female during mid‐pregnancy (Hudson & Browman, 1959; Short, 1970; Thorne, Dean, & Hepworth, 1976). Thus, higher survival was exhibited by neonates born to mothers that likely had greater access to high quality and quantity of forage in mid‐to‐late pregnancy. Additionally, the nutritional intake by a pregnant female is directly related to the size of a neonate at birth and is considered a form of maternal allocation (Parker et al., 2009). Therefore, maternal females that were on a higher nutritional plane and were subjected to higher quality forage during gestation generally give birth to larger young. Our data also indicated that size of a neonate at birth positively influenced survival, and thus, could potentially be used as an index of its nutritional condition and the forage quality acquired by the mother during mid to late gestation. Those young that were born larger from females that may have had access to high quality resources exhibited higher survival to recruitment and thus, had a greater effect on the population performance in that ecosystem.

Normalized difference in vegetation index values during February to May for the areas occupied by pregnant females were most indicative of survival of young; however, we did not find an effect of rump fat or body condition score (BCS) of the maternal female on estimated neonatal survival. Maternal nutritional plane has a large influence on juvenile survival, but our measurements were taken early in gestation (February–March). Thus, measurements of nutritional condition during late pregnancy and near parturition, which we have shown to strongly influence survival, could not be directly measured because of the timing of our captures. Conversely, Monteith et al. (2014) found an effect of body condition in mid‐March on juvenile survival and recruitment, but animals in that region may not have access to rapid and short‐term pulse of term green‐up that is frequently observed after rainfall events in desert regions as exhibited in our study area. Moreover, relative condition of the female earlier during gestation may be a good measure of reproductive output in cooler, wetter environments or for migratory populations compared to arid regions where plant responses to rainfall are rapid, such as in our study. Indeed, our initial models indicated that NDVI measured February through May had the strongest effect on survival of young. If forage quality through May affected size of young, our measurements of nutritional condition of the mothers in February or early March, would have been much lower prior to those females experiencing high quality increase in forage between our captures and parturition.

Neither body weight of neonates nor our index of body condition was supported in our top survival models. Given that neonatal mule deer have no subcutaneous fat and limited visceral fat, relative timing of nursing events may have a strong influence on measured weight at the time of handling (Short, 1970). As a result, residuals from our regression may not be an accurate index of body condition of neonates. Quintana et al. (2016) investigated survival of young in an arid ecosystem, and similar to our results, they showed evidence that structural size, not weight or condition, was indicative of survival, although their confidence intervals overlapped zero. Further, our findings indicate that environmental factors affecting the mother's forage quality and fetal development (i.e., larger neonates) are the strongest indicators of survival of young. Moreover, our measures of landscape characteristics of the birth site and habitat characteristics the female chose postparturition (Table 1) did not strongly influence survival of neonates because important factors leading to probability of survival may have already been established prior to birth.

Spring rains and associated green‐up can heighten the nutritional plane for individuals after migration events in temperate regions and can also be crucial for large herbivores entering the reproductive period (Parker et al., 2009). Additionally, late‐summer precipitation and autumn green‐up can play a major role in body condition and fecundity for ungulate populations entering the breeding season, and overwinter survival thereafter (Cook et al., 2004; Stewart, Bowyer, Dick, Johnson, & Kie, 2005; Tollefson, Shipley, Myers, Keisler, & Dasgupta, 2010). Measures of NDVI have been shown to be reliable indices of timing and amount of green‐up relative to the influences on population‐level responses in large herbivores (Borowik, Pettorelli, Sönnichsen, & Jędrzejewska, 2013; Creech, Epps, Monello, & Wehausen, 2016; Hamel, Garel, Festa‐Bianchet, Gaillard, & Côté, 2009). Previous literature on the ecology of large herbivores has shown that behavioral, decision‐based movement of large ungulates can be explicitly tied to timing and amount of green‐up as evidenced by NDVI (Sawyer & Kauffman, 2011). Further, demographic responses such as adult survival of mule deer have been tied to measures of nutritional resources including NDVI (Hurley et al., 2014).

Survival of adult females within large herbivore populations has the potential to affect population growth rate, but survival of adults is generally is high and tends to vary little in most populations (Gaillard & Yoccoz, 2003; Hurley et al., 2011; Unsworth, Pac, White, & Bartmann, 1999). Conversely, survival and recruitment of young are highly variable, which can strongly affect population growth (Gaillard et al., 1998). Few studies, however, have directly linked measures of NDVI and effects of maternal condition on early survival of young, especially in arid ecosystems. Hurley et al. (2014) evaluated the direct relationship between NDVI (available forage in spring and autumn) with overwinter survival of young and reported that autumn NDVI values strongly predicted overwinter survival. Nevertheless, spring green‐up had little effect on survival in that ecosystem and indicates the possible transition from maternal condition passed on to young in early life stages and later; the direct effects of available forage after young are weaned. In our ecosystem, however, animals were not subjected to harsh winters with extreme temperatures or deep snow and their associated constraints on access to forage. Nevertheless, our study animals were subjected to high temperatures and low precipitation that characterize hot, dry regions, during summer. Therefore, large herbivores occupying an arid environment may be more reliant on increased forage quality during gestation that is allocated to the offspring rather than relying on nutritional sources postpartum to support young to recruitment. Females also experience the most nutritionally demanding period of their life cycle during lactation (Clutton‐Brock, Albon, & Guinness, 1989), which coincides with high temperatures and low precipitation in our study ecosystem. Thus, nutritional sources that female is exposed to mid‐pregnancy would be better suited to allocating to development of young thereby increasing the probability of survival and increasing maternal fitness.

Arid regions of the world are expected to become hotter and dryer with less predictable pulses of rainfall (Mahlman, 1997; Meehl et al., 2000). Hence, climate change has the potential to substantially alter population dynamics of large herbivores occupying these arid ecosystems. We have identified and quantified a potential link between maternal nutritional acquisition during pregnancy and survival of young. Precipitation events that lead to increases in forage quality and availability during mid‐ to late‐pregnancy may prove to be the optimum timeframe for acquiring and allocating nutrients to young in arid environments. As droughts become more frequent and severe, large herbivore populations and all other biota likely will have to adjust to these altered climatic conditions. If rainfall patterns shift and spring rainfall events become less common in arid regions, populations may be forced to temporally shift their reproductive cycle or develop new strategies for resource acquisition. The inability to do so could drastically affect population performance of large herbivores and potentially other species that are reliant on landscape resources in desert regions. Understanding the link between environmental patterns and population performance has vast implications for comprehending how our natural ecosystems will react to the expected shifts in climate throughout our natural world.

## CONFLICT OF INTERESTS

None declared.

## AUTHOR CONTRIBUTIONS

KMS, VCB, and NWD obtained funding and oversaw the project. LJH and APB collected data for the project in the field and LJH analyzed the data with the assistance of JSS. LJH interpreted the results and wrote the manuscript with help from KMS, APB, JSS, NWD, and VCB. All authors provided editorial assistance.
